# Expedited pathway portfolios, trial complexity, and FDA approval timelines in oncology and neurology: A comparative analysis

**DOI:** 10.1371/journal.pone.0355495

**Published:** 2026-08-27

**Authors:** Ahmed M. Sayed, Nada M. Ezzelarab, Nguyen Tien Huy

**Affiliations:** 1 Department of Chemistry, College of Science, Purdue University, West Lafayette, Indiana, United States of America; 2 Department of Biology, School of Sciences and Engineering, The American University in Cairo, Cairo, Egypt; 3 Institute of Research and Development, Duy Tan University, Da Nang, Vietnam; 4 School of Medicine and Pharmacy, Duy Tan University, Da Nang, Vietnam; Society for Brain Mapping and Therapeutics, UNITED STATES OF AMERICA

## Abstract

FDA expedited programs are intended to facilitate development and review for therapies addressing serious conditions with unmet medical need, but their use may depend on whether the evidentiary package is compatible with regulatory acceleration. We compared FDA original approvals in oncology and neurology from 2015 to 2024 to evaluate whether differences in approval timing reflected expedited pathway portfolio intensity, pivotal evidence architecture, or alignment between the two. We conducted a retrospective comparative cohort study of 195 original FDA approvals, including 136 oncology products and 59 neurology products. Product-level regulatory data were curated from FDA approval materials. Pivotal or registrational trials supporting first approval were identified from FDA labels and review documents and enriched through exact National Clinical Trial identifier matching to AACT. The final evidence dataset included 221 curated product–trial links. Regulatory pathway-portfolio intensity was summarized using a Regulatory Bundling Index incorporating Fast Track, Breakthrough Therapy designation, Priority Review, Accelerated Approval, and Orphan designation. Outcomes included regulatory approval time, clinical development time, total time to approval, and pivotal evidence architecture. Oncology products had higher pathway intensity than neurology products, with median Regulatory Bundling Index 3 [IQR, 2–4] versus 2 [IQR, 0–3] (p < 0.001). Neurology products had longer regulatory approval time than oncology products: 362 days [IQR, 245–366] versus 234 days [IQR, 182–329] (p < 0.001). In contrast, clinical development time and total time to approval did not differ significantly. Neurology pivotal evidence was more often randomized, masked, and late phase, while oncology evidence was more often single group. Trial enrollment did not differ significantly. In exploratory counterfactual analyses, increasing neurology pathway intensity to the oncology median was associated with a median predicted review-time reduction of 37.8 days. Differences in FDA approval timing between oncology and neurology appear to reflect both pathway configuration and pivotal evidence architecture. These findings support a pathway-evidence alignment framework rather than a simple model in which more expedited designations alone explain faster approval.

## Introduction

FDA expedited programs sit at the center of a persistent regulatory tension: patients with serious diseases need earlier access to promising therapies, but earlier access often comes with greater evidentiary uncertainty. The FDA has established four major expedited programs for drugs and biologics—Fast Track, Breakthrough Therapy designation, Accelerated Approval, and Priority Review—to facilitate development and review for serious conditions with unmet medical need [1]. Their use has expanded over time [2,3]. Yet acceleration is not a uniform regulatory event. A designation may shorten review, intensify FDA-sponsor interaction, permit reliance on a surrogate endpoint, or signal a serious-disease context. Its practical effect depends on the disease, the endpoint, and the pivotal evidence package.

This issue is especially visible in oncology. Cancer drug development has often been a proving ground for accelerated and expedited regulatory strategies, supported by biomarker-defined populations, response-based endpoints, rare molecular subgroups, and single-group or open-label evidence in selected settings [4,5]. These features can make earlier regulatory decision-making feasible. They also create uncertainty. Prior studies have shown substantial variation in the pivotal evidence supporting FDA approvals, including differences in trial number, randomization, masking, comparator use, endpoint selection, enrollment, and follow-up [5]. Accelerated Approval remains particularly debated because earlier approval may rely on surrogate or intermediate endpoints, while confirmatory studies are needed to verify clinical benefit [6,7].

Neurology presents a different problem. Many neurological diseases are serious, disabling, and high-need, but their evidence packages often depend on functional outcomes, longer follow-up, heterogeneous disease progression, and clinical outcome assessments that may be difficult to validate or interpret. A neurological product may therefore require more controlled and time-intensive evidence even when the disease burden is high. This makes oncology and neurology a useful comparison. Both fields include serious diseases and can qualify for expedited programs, but the scientific conditions for regulatory acceleration may differ.

Recent cross-therapeutic evidence supports this disease-context view. In a decadal review of infectious-disease approvals, expedited-program use differed between infectious and non-infectious products, with greater use of Fast Track and Priority Review but less use of Accelerated Approval in infectious diseases [8]. That pattern suggests that expedited pathways are shaped not only by urgency, but also by endpoint structure and regulatory fit.

What remains less clear is how pathway intensity and pivotal evidence architecture interact. Most prior work has evaluated expedited programs individually or described approval evidence without linking it to bundled pathway use. In practice, products may receive several designations together. To address this gap, we studied FDA original oncology and neurology approvals from 2015 to 2024 using FDA-source-anchored pivotal-evidence curation and exact NCT enrichment through AACT. We summarized pathway portfolio intensity using the Regulatory Bundling Index and compared it with approval timelines and pivotal evidence architecture. The novelty of this study is an integrated pathway-evidence framework: it asks whether review-time differences reflect regulatory configuration alone, evidentiary burden alone, or the alignment between the two.

## Methods

### Study design and reporting

We conducted a retrospective comparative cohort study of original U.S. Food and Drug Administration (FDA) approvals in oncology and neurology from January 1, 2015, through December 31, 2024. The regulatory database was accessed on August 1, 2025. The study used only publicly available regulatory and trial-registry data and did not involve individual-level patient data, direct participant contact, intervention, or identifiable private information; therefore, institutional review board approval was not required. Reporting was guided by the STROBE recommendations for observational studies.

### Eligibility criteria

Eligible products were original FDA approvals assigned to oncology or neurology at first approval. Products were included if they were approved as new molecular entities under a New Drug Application or as original Biologics License Applications. Supplemental approvals, generic products, biosimilars, reformulations, route or dosage-form changes without a new active entity, and products regulated solely as devices or diagnostics were excluded. Therapeutic area was assigned using the primary FDA-approved indication at first approval, supported by FDA approval packages and product labels.

### Data sources and regulatory curation

Product-level regulatory data were obtained from Drugs@FDA, FDA approval letters, product labels, and FDA review documents. Extracted variables included product name, application type, approval date, NDA/BLA submission date when available, therapeutic area, and regulatory designations. The designations abstracted were Fast Track, Breakthrough Therapy designation, Priority Review, Accelerated Approval, and Orphan designation. The final product-level cohort included 195 original approvals: 136 oncology products and 59 neurology products. Product-level analyses used all 195 approvals, including products without a printed valid NCT identifier when approval information was otherwise source-verified.

### Pivotal-trial identification and exact NCT enrichment

Pivotal or registrational evidence was identified from FDA labels and review materials. The goal was to capture trials supporting first FDA approval, not all trials associated with a product. NCT identifiers were extracted only when they were printed in FDA source materials and could be manually verified. Products with FDA-described pivotal evidence but no printed valid NCT identifier were retained in product-level analyses but excluded from NCT-enriched trial-metadata analyses. Curated NCT identifiers were enriched through exact matching to AACT, the relational database derived from ClinicalTrials.gov. No fuzzy matching, product-name-only matching, or broad ClinicalTrials.gov search matching was used. The final curated evidence dataset contained 221 product–NCT pivotal links, representing 220 distinct NCT identifiers; all 221 links were matched exactly to AACT.

### Units of analysis

Two analytic levels were used. Product-level analyses used the regulatory application as the unit of analysis and included all 195 approvals. These analyses evaluated regulatory designations, Regulatory Bundling Index, approval timelines, and the number of curated pivotal NCT links per product. Product–NCT link-level analyses used each curated pivotal product–NCT link as the unit of analysis and were restricted to the 221 exact NCT-enriched pivotal links. These analyses evaluated evidence architecture, including allocation, masking, phase, intervention model, enrollment, and exploratory design complexity.

### Outcomes

The primary timing outcome was regulatory approval time (RAT), defined as the number of calendar days from NDA/BLA submission to FDA approval. Clinical development time (CDT) was defined as the interval from the earliest stable first-in-human or first clinical-development anchor date to NDA/BLA submission. Total time to approval (TTA) was defined as CDT plus RAT. Date anchors were selected using prespecified precedence rules. Products without a stable anchor date for a specific interval were excluded only from analyses requiring that interval. RAT was reported in days; CDT and TTA were reported in years.

### Exposures and evidence-architecture variables

The primary regulatory exposure was expedited pathway portfolio intensity, summarized by the Regulatory Bundling Index (RBI). RBI was calculated as the sum of five binary indicators: Fast Track, Breakthrough Therapy designation, Priority Review, Accelerated Approval, and Orphan designation. Scores ranged from 0 to 5, with higher values indicating a greater number of expedited or orphan designations. Orphan designation was included because it reflects an important regulatory-development context and frequently co-occurs with FDA expedited programs, although it is not itself one of the four FDA expedited programs for serious conditions.

Evidence-architecture variables were extracted from exact AACT-enriched NCT records. Allocation was classified as randomized or not randomized. Masking was classified as masked or not masked. Phase 3 and phase 2/3 studies were classified as late phase. Intervention model was used to identify single-group designs. Enrollment was analyzed using the AACT-reported enrollment value. An exploratory design-complexity score summarized structured trial features, including randomized allocation, masking, late-phase status, non-single-group design, and enrollment scale. The enrollment component was scaled as min(log10(enrollment + 1) / 4, 1) to reduce the influence of very large trials. The score was used descriptively and was not treated as a validated clinical-development burden instrument. Score components are described in Table I in S1 File.

### Statistical analysis

Continuous and ordinal variables were summarized as medians and interquartile ranges. Categorical variables were summarized as counts, denominators, and percentages. Oncology and neurology were compared using Wilcoxon rank-sum tests for continuous or ordinal variables and Fisher exact tests for categorical variables. For major binary evidence-architecture features, absolute percentage-point differences were calculated as neurology minus oncology and reported with 95% confidence intervals. All P values were two-sided. Exact P values were reported when P ≥ 0.001, and smaller values were reported as P < 0.001.

The primary analyses were descriptive and comparative. They were designed to quantify between-area differences in pathway intensity, approval timelines, and pivotal evidence architecture. No causal effect of therapeutic area or designation status was assumed. Missing data were handled using complete-case analysis for the variable or interval being evaluated. Products without valid curated NCT identifiers were retained in product-level analyses but excluded from NCT-enriched evidence-architecture analyses.

### Joint pathway-evidence, counterfactual, and sensitivity analyses

To explore whether pathway intensity and evidence architecture jointly structured review time, product-level evidence complexity and RBI were grouped into prespecified strata, and median RAT was summarized within populated therapeutic-area strata. Cell counts were reported, and low-count cells were interpreted cautiously.

Exploratory counterfactual analyses estimated how predicted neurology RAT might change under higher pathway-intensity scenarios. A product-level log-linear model was fit using log-transformed RAT as the outcome and therapeutic area, RBI, evidence complexity, log enrollment, and curated NCT-link count as predictors. The primary scenario raised neurology products with RBI below the oncology median to RBI 3; a sensitivity scenario raised eligible neurology products to RBI 4. Predicted RAT values were back-transformed to days, and bootstrap resampling was used to summarize uncertainty. Economic translation used prespecified assumptions of $1.0 million per RAT day and $0.5 million per CDT day, with CDT held constant.

Robustness analyses repeated the RAT comparison in the full cohort, the valid-NCT subset, after excluding the top 1% of RAT values, and after winsorizing RAT. A supplementary gradient-boosted model was fit only as a predictive sensitivity analysis and was not used for primary inference. Analyses were conducted in R.

## Results

### A conservative FDA-source-anchored cohort with exact pivotal-trial linkage

The final curated cohort included 195 original FDA approvals from 2015–2024, comprising 136 oncology products and 59 neurology products (Table 1). Of these, 174 products had at least one valid curated NCT identifier linked to pivotal or registrational evidence supporting first FDA approval, whereas 21 products had FDA label- or review-verified pivotal evidence without a printed valid NCT identifier. These 21 products were retained in product-level regulatory analyses but excluded from NCT-based trial-metadata summaries. Fig A in S1 File summarizes the linkage audit: the curated evidence set contained 221 product–NCT pivotal links, representing 220 distinct NCT identifiers, and all 221 links were enriched by exact AACT matching. The corresponding denominator structure is provided in Table A in S1 File. Thus, the full product-level analyses used 195 approvals, while product–NCT link-level analyses used the 221 verified pivotal links.

**Table 1:** Product-level regulatory characteristics and approval timelines by therapeutic area.

Characteristic	Neurology (n = 59)	Oncology (n = 136)	p-value
**Products with valid curated NCT, n (%)**	49 (83.1)	125 (91.9)	0.080
**Products without valid curated NCT, n (%)**	10 (16.9)	11 (8.1)	—
**Fast Track, n (%)**	24 (40.7)	56 (41.2)	1.000
**Breakthrough Therapy designation, n (%)**	12 (20.3)	69 (50.7)	<0.001
**Priority Review, n (%)**	28 (47.5)	113 (83.1)	<0.001
**Accelerated Approval, n (%)**	6 (10.2)	58 (42.6)	<0.001
**Orphan designation, n (%)**	29 (49.2)	98 (72.1)	0.003
**Regulatory Bundling Index, median [IQR]**	2 [0–3]	3 [2–4]	<0.001
**RBI = 0, n (%)**	23 (39.0)	8 (5.9)	<0.001
RBI = 4–5, n (%)	7 (11.9)	53 (39.0)	<0.001
**Regulatory approval time, days, median [IQR]**	362 [245–366]	234 [182–329]	<0.001
**Clinical development time, years, median [IQR]**	6.51 [4.73–11.19]	6.43 [4.61–9.19]	0.703
**Total time to approval, years, median [IQR]**	7.48 [5.29–12.08]	7.29 [5.18–9.89]	0.413
**Curated pivotal NCT links per product, median [IQR]**	1 [1–2]	1 [1–1]	0.014

### Oncology carried higher pathway intensity and shorter regulatory review time

**Fig 1:**
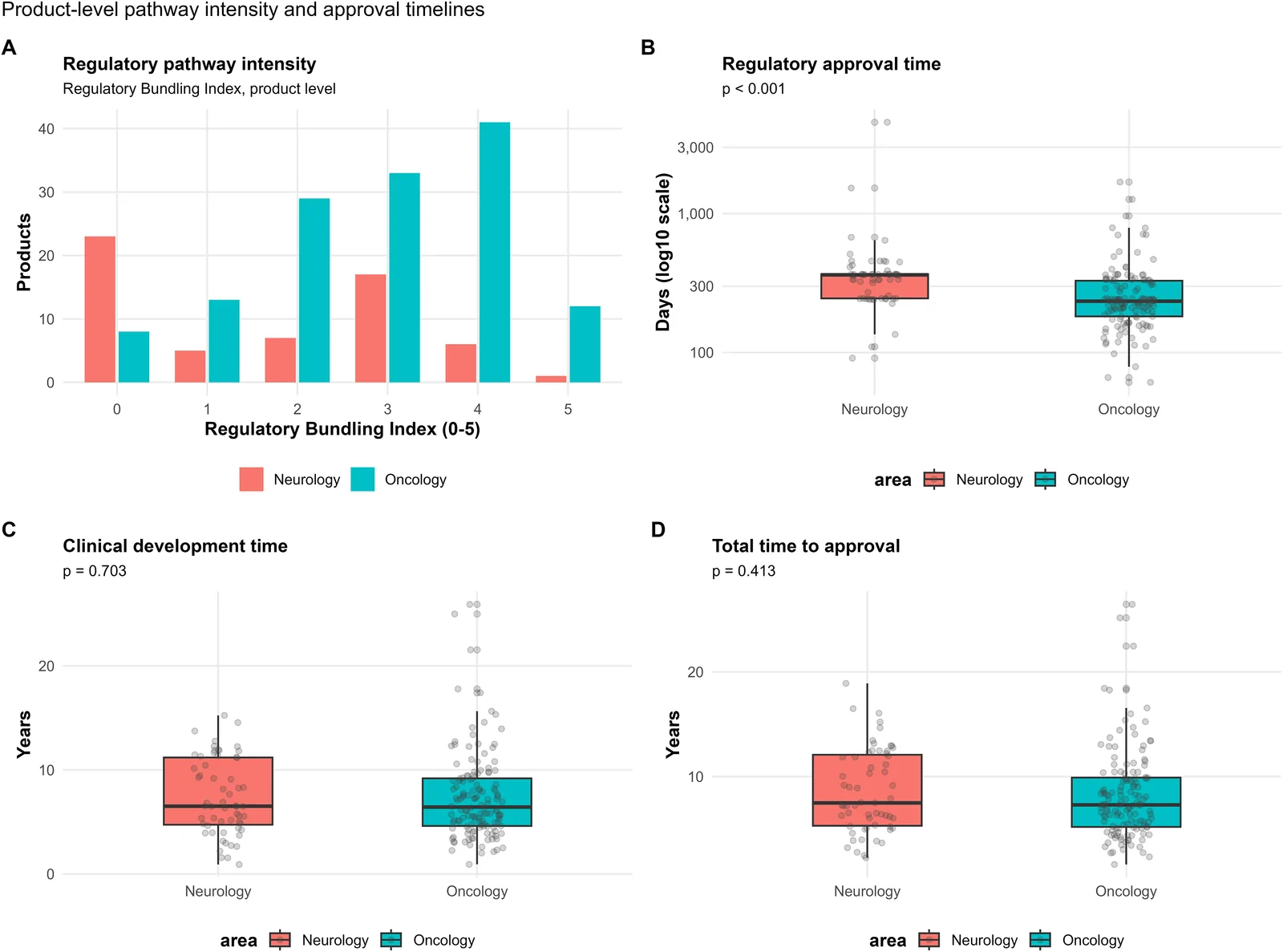
Product-level pathway intensity and approval timelines; A: Regulatory Bundling Index distribution by area; B: Regulatory approval time, RAT; C: Clinical development time, CDT; D: Total time to approval, TTA.

Figure 1A shows the product-level distribution of the Regulatory Bundling Index (RBI). Oncology products were concentrated in higher pathway-intensity strata, with a median RBI of 3 [IQR, 2–4], compared with 2 [IQR, 0–3] in neurology (p < 0.001; Table 1). High-intensity pathway portfolios, defined as RBI 4–5, occurred in 53 of 136 oncology products but in only 7 of 59 neurology products. Conversely, RBI 0 was observed in 23 neurology products and 8 oncology products. This distribution reflected greater oncology use of Priority Review, Breakthrough Therapy designation, Accelerated Approval, and Orphan designation, while Fast Track use was similar between areas.

Regulatory approval time (RAT) showed a parallel separation. In Fig 1B, neurology products had longer review intervals than oncology products, with median RAT of 362 days [IQR, 245–366] versus 234 days [IQR, 182–328.5], respectively (p < 0.001; Table 1). However, broader development intervals were not significantly different. Median clinical development time was 6.51 years [IQR, 4.73–11.19] in neurology and 6.43 years [IQR, 4.61–9.19] in oncology (p = 0.703; Fig 1C). Similarly, total time to approval was 7.48 years [IQR, 5.29–12.08] versus 7.29 years [IQR, 5.18–9.89] (p = 0.413; Fig 1D). Together, Fig 1 and Table 1 indicate that the clearest timing difference occurred during FDA review rather than across the full clinical-development interval.

### Curated pivotal evidence architecture differed sharply between areas

The curated pivotal-evidence dataset contained 142 oncology product–NCT links and 79 neurology links (Table 2). Although the median number of curated pivotal NCT links per product was 1 in both therapeutic areas, the distributions differed modestly: neurology products had 1 link per product [IQR, 1–2], compared with 1 [IQR, 1–1] in oncology (p = 0.014; Table 1). The standalone distribution is shown in Fig C in S1 File.

**Table 2:** Curated pivotal evidence architecture by therapeutic area.

Characteristic	Neurology links (n = 79)	Oncology links (n = 142)	Difference or p-value
**Product–NCT pivotal links, n**	79	142	—
**Distinct NCT identifiers, n**	79	141	—
**Randomized, n/N (%)**	77/79 (97.5)	107/142 (75.4)	+22.1 pp [14.2 to 30.0]; p < 0.001
**Masked, n/N (%)**	75/79 (94.9)	35/142 (24.6)	+70.3 pp [61.7 to 78.9]; p < 0.001
**Late phase, n/N (%)**	65/79 (82.3)	60/142 (42.3)	+40.0 pp [28.3 to 51.7]; p < 0.001
**Single-group design, n/N (%)**	2/79 (2.5)	37/142 (26.1)	−23.5 pp [−31.5 to −15.5]; p < 0.001
**Enrollment, median [IQR]**	427 [172–979]	329 [196–559]	p = 0.152
**Design-complexity score, median [IQR]**	4.62 [4.49–4.75]	2.68 [1.59–3.73]	p < 0.001

**Fig 2:**
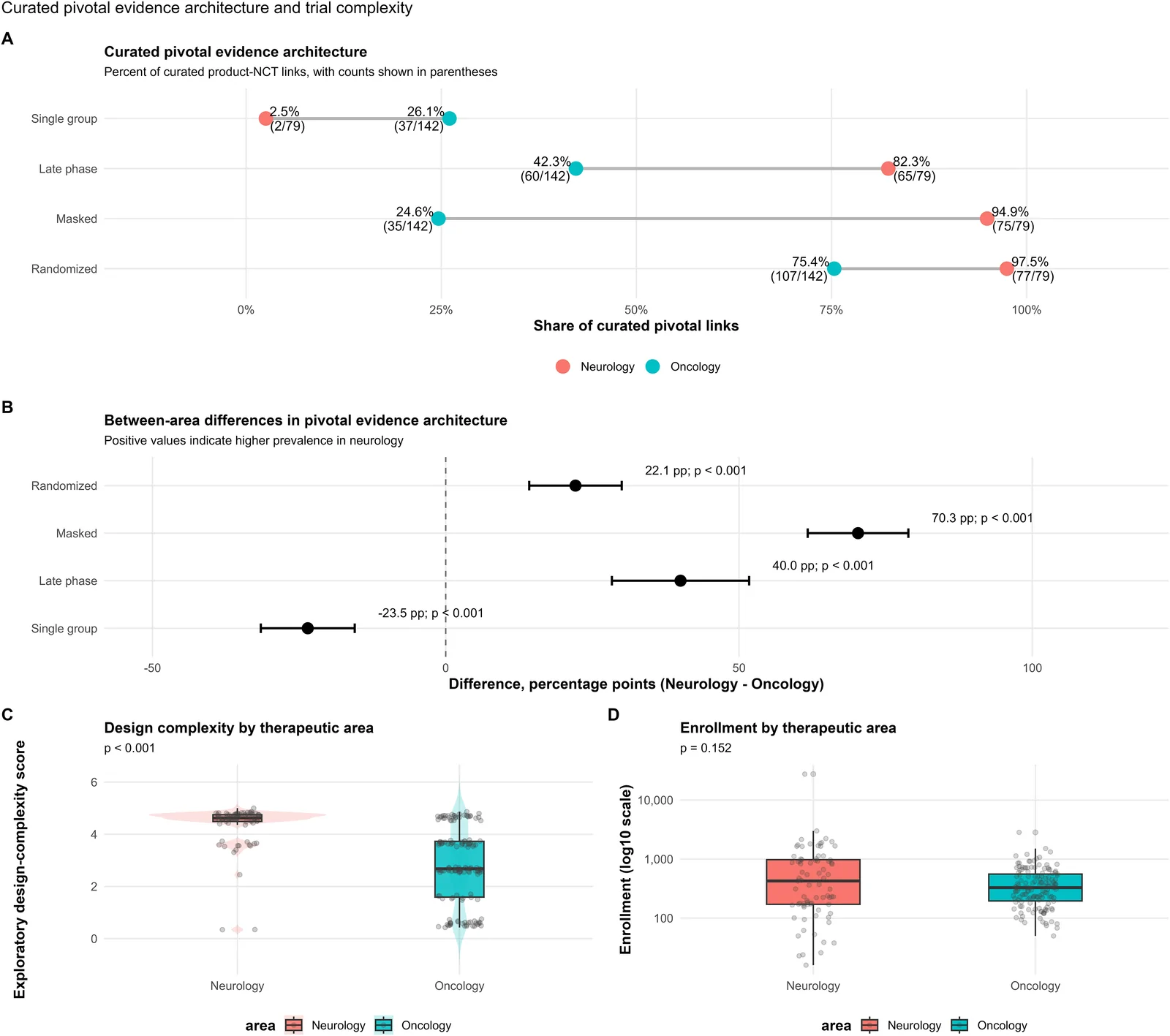
Curated pivotal evidence architecture and trial complexity; A: Randomized / masked / late-phase / single-group proportions; B: Between-area percentage-point differences with 95% CIs; C: Design-complexity score; D: Enrollment distribution.

Figure 2A displays the major evidence-architecture features. Neurology pivotal links were more likely to be randomized, masked, and late-phase. Randomized allocation occurred in 77 of 79 neurology links (97.5%) and 107 of 142 oncology links (75.4%), giving a neurology-minus-oncology difference of +22.1 percentage points (95% CI, 14.2 to 30.0; p < 0.001; Fig 2B; Table C in S1 File). Masking showed the largest absolute separation: 75 of 79 neurology links (94.9%) were masked, compared with 35 of 142 oncology links (24.6%), a +70.3 percentage-point difference (95% CI, 61.7 to 78.9; p < 0.001). Late-phase evidence was also more common in neurology (65/79, 82.3%) than oncology (60/142, 42.3%), corresponding to a +40.0 percentage-point difference (p < 0.001). By contrast, single-group evidence was more frequent in oncology (37/142, 26.1%) than neurology (2/79, 2.5%), producing a −23.5 percentage-point difference (p < 0.001). Additional allocation, masking, phase, and intervention-model distributions are shown in B in S1 File, with statistical tests summarized in Table B in S1 File.

### Evidence architecture, more than enrollment, explained the pivotal-trial contrast

The evidence-architecture contrast was captured by the exploratory design-complexity score. Fig 2C shows that neurology pivotal links had higher complexity scores than oncology links: median 4.62 [IQR, 4.49–4.75] versus 2.68 [IQR, 1.59–3.73], respectively (p < 0.001; Table 2). This higher neurology score was consistent with the greater frequency of randomized, masked, and late-phase designs. In contrast, trial enrollment was not significantly different. Median enrollment was 427 participants [IQR, 171.5–978.5] for neurology links and 328.5 [IQR, 196.0–558.5] for oncology links (p = 0.152; Fig 2D; Table 2). Thus, the main evidence difference was architectural rather than simply a difference in trial scale.

### A joint pathway-evidence map separated oncology and neurology timing landscapes

**Fig 3:**
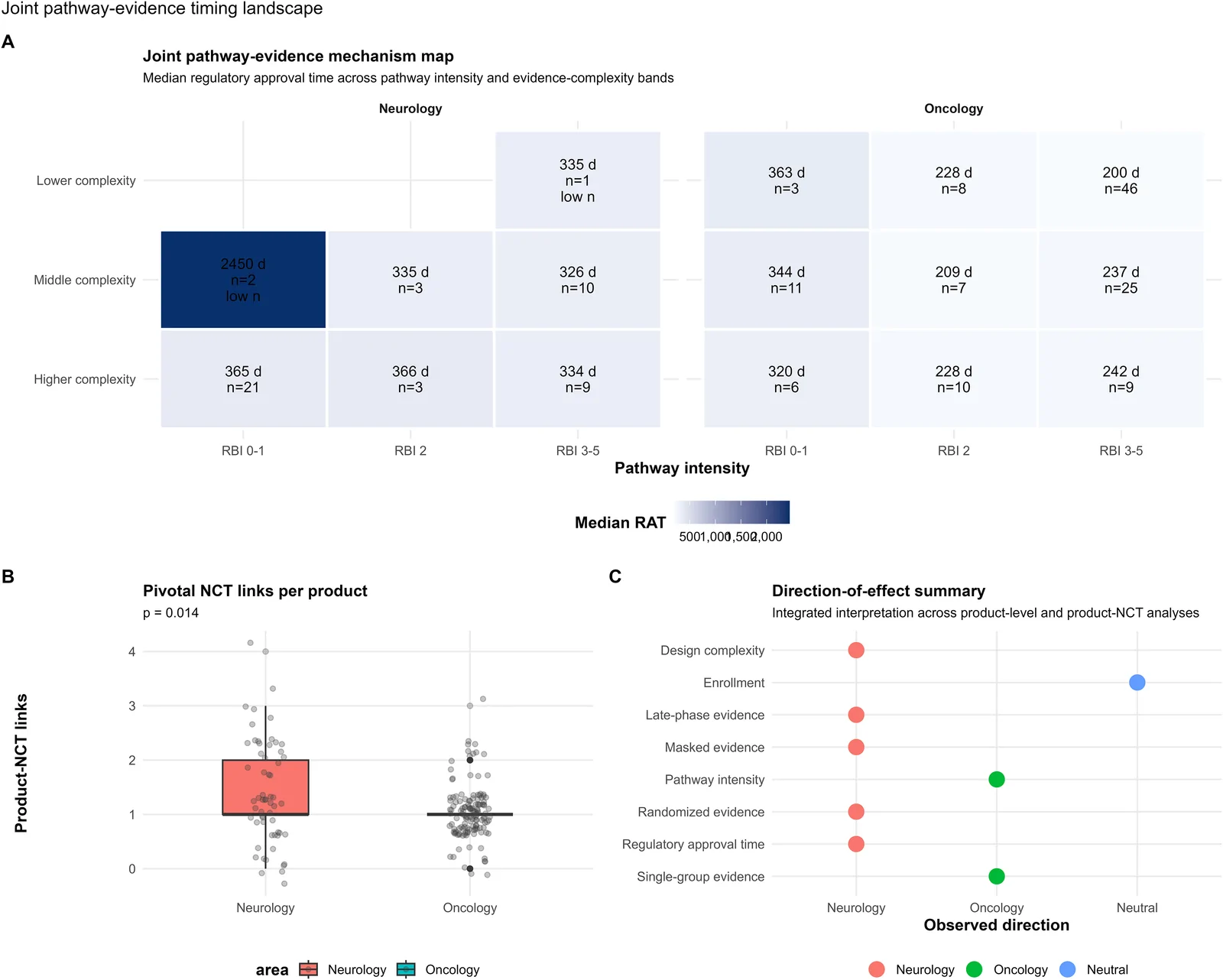
Joint pathway-evidence timing landscape; A: RBI × evidence-complexity heatmap with median RAT; B: Curated pivotal NCT links per product; C: Direction-of-effect summary.

Figure 3A integrates RBI, evidence-complexity bands, and median RAT. Oncology products were more often located in high-RBI strata, whereas neurology products were more often located in lower or intermediate RBI strata with higher evidence complexity. Across populated neurology strata, median RAT ranged from 326.5 to 2449.5 days, although the highest value occurred in a low-count cell with two products and should be interpreted cautiously. In oncology, median RAT ranged from 200.5 to 363.0 days. The shortest median RAT was observed in the RBI 3–5/lower-complexity oncology cell, where 46 products had a median RAT of 200.5 days. Full cell-level values are reported in Table D in S1 File. Meanwhile, Fig 3B shows the distribution of curated pivotal NCT links per product, with neurology products having a median of 1 [IQR, 1–2] link compared with 1 [IQR, 1–1] in oncology (p = 0.014). Lastly, Fig 3C summarizes the integrated direction of effects, supporting a joint pathway-evidence interpretation rather than a single-factor explanation.

### Exploratory pathway intensification predicted shorter neurology review time

**Fig 4:**
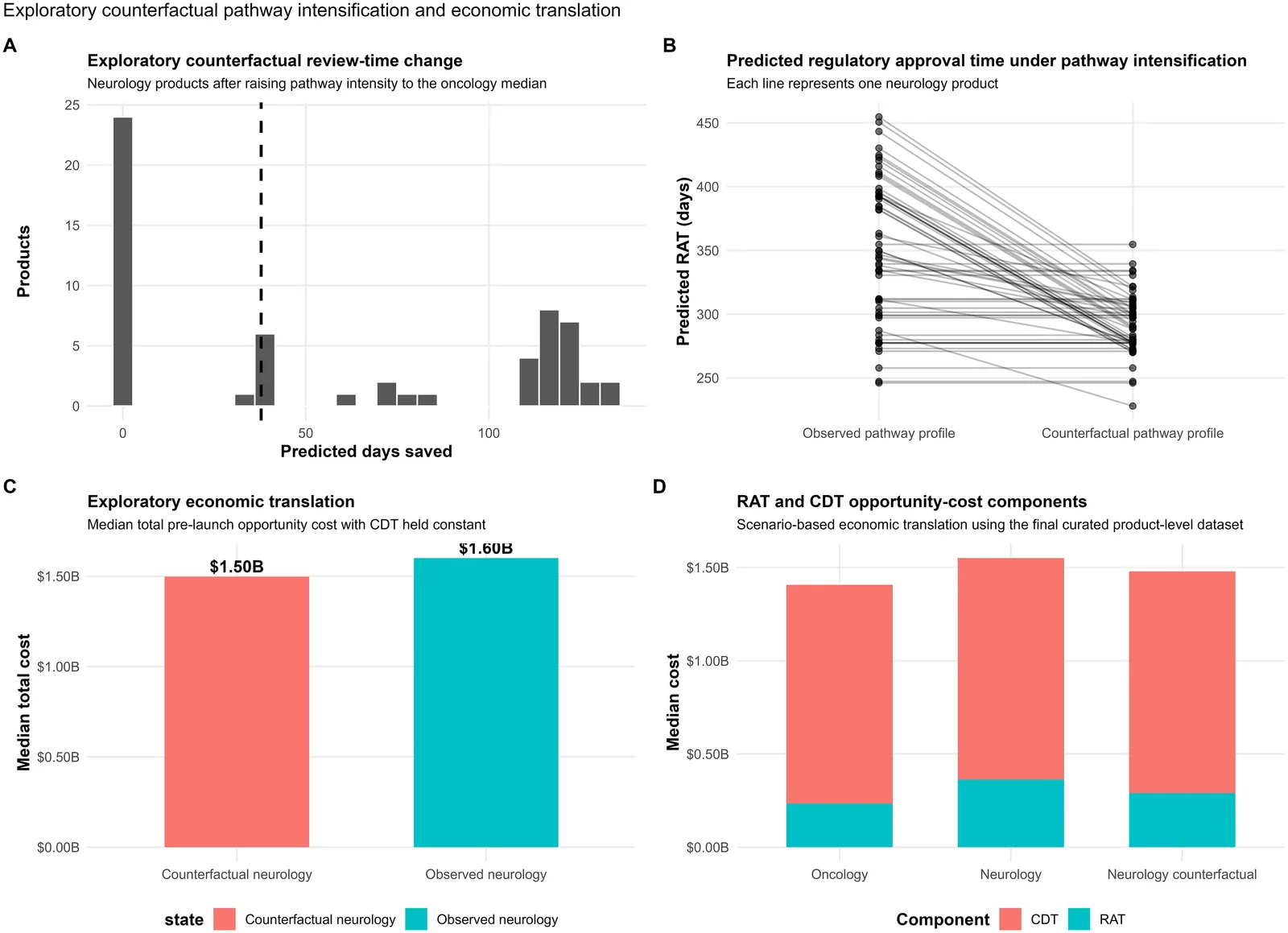
Exploratory counterfactual pathway intensification and economic translation; A: Predicted days saved under RBI-to-oncology-median scenario; B: Paired observed vs counterfactual predicted RAT; C: Median total pre-launch opportunity cost observed vs counterfactual; D: RAT/CDT opportunity-cost components.

To assess whether lower pathway intensity could partly explain longer neurology review times, we performed an exploratory counterfactual simulation. In the primary scenario, neurology products with RBI below the oncology median were reassigned to RBI 3, while preserving therapeutic area, evidence-complexity measures, enrollment summaries, and curated NCT-link structure. Table 3 summarizes this scenario, and Fig 4A visualizes the distribution of predicted days saved. Median predicted neurology RAT decreased from 346.6 to 289.8 days, corresponding to a median reduction of 37.8 days [IQR, 0.0–115.0]. Bootstrap analysis yielded a median reduction of 38.1 days, with a 95% interval of 22.1 to 55.5 days. Fig 4B shows paired product-level predicted RAT values, with additional paired-prediction outputs in Fig E in S1 File.

**Table 3:** Exploratory counterfactual and economic translation for neurology products.

Scenario	Observed predicted RAT, days	Counterfactual predicted RAT, days	Median days saved [IQR]	Bootstrap median days saved [95% interval]	Median RAT-cost saving	Median total-cost saving
**Raise neurology RBI to oncology median (RBI = 3)**	346.6	289.8	37.8 [0.0–115.0]	38.1 [22.1–55.5]	$37.8M	$37.8M
**Raise neurology RBI to oncology upper quartile (RBI = 4)**	346.6	262.2	71.5 [32.8–145.3]	70.7 [40.8–101.7]	$71.5M	$71.5M

A more intensive scenario raised eligible neurology products to RBI 4. In that scenario, median predicted RAT decreased from 346.6 to 262.2 days, corresponding to a median reduction of 71.5 days [IQR, 32.8–145.3]. The bootstrap median reduction was 70.7 days, with a 95% interval of 40.8 to 101.7 days. Detailed model fit, coefficients, bootstrap results, and product-level counterfactual predictions are provided in Table E in S1 File. The model was statistically significant overall (R² = 0.191; adjusted R² = 0.170; p < 0.001), and higher RBI was associated with shorter log-transformed RAT after preserving therapeutic area and evidence-architecture covariates.

### Economic translation showed interpretable but scenario-based opportunity-cost reductions

Using prespecified assumptions of $1.0 million per RAT day and $0.5 million per clinical-development day, the primary counterfactual scenario reduced median predicted neurology RAT-related opportunity cost from $346.6M to $289.8M (Fig 4C; Table 3). With clinical-development cost held constant, median total pre-launch opportunity cost decreased from $1.60B to $1.50B, corresponding to a median saving of $37.8M. Under the RBI 4 scenario, the median saving increased to $71.5M. Fig 4D separates RAT and clinical-development cost components, and Table F in S1 File provides the full economic translation, including observed economic context by therapeutic area. These estimates are scenario-based translations of modeled timing differences, not causal estimates of realized savings.

### Robustness and predictive sensitivity analyses supported a descriptive inference strategy

The primary RAT difference remained significant in the full cohort, in the NCT-linked product subset, after excluding the top 1% of RAT values, and after winsorizing RAT values (all p < 0.001; Fig D in S1 File; Table H in S1 File). A supplementary gradient-boosted model was also fit as a predictive sensitivity analysis, but it was not used for primary inference. Out-of-fold performance was limited (R² = 0.029 on the log-RAT scale; RMSE = 357.5 days; MAE = 137.2 days), supporting the decision to emphasize conservative curation, transparent descriptive inference, joint pathway-evidence mapping, and scenario-based counterfactual translation in the main analysis (Fig F in S1 File; Table G in S1 File).

## Discussion

This comparative analysis of FDA oncology and neurology approvals from 2015 to 2024 shows that regulatory review time differed more clearly than overall development time. Oncology products had higher expedited-pathway portfolio intensity and shorter regulatory approval times. Neurology products had lower pathway intensity, longer review intervals, and more conservative pivotal evidence architecture. The central finding is therefore not that one therapeutic area is simply “faster” than the other. Rather, review speed appears to reflect how regulatory pathways, endpoint maturity, and pivotal-trial design fit together.

The revised analysis also addresses an important methodological concern. The pivotal-trial dataset was rebuilt around FDA-source-anchored manual curation rather than broad product-name matching in ClinicalTrials.gov. AACT was then used only for exact-NCT metadata enrichment. This matters for interpretation. A broad trial-linkage strategy can inflate the apparent evidence base by capturing supportive, historical, off-label, or unrelated trials. By contrast, the revised dataset focuses on pivotal or registrational evidence supporting first FDA approval. This makes the evidence-architecture comparison narrower, but more defensible.

Our findings extend prior work on expedited programs. Earlier studies have shown that FDA expedited designations have become increasingly common, but they have also raised questions about whether all accelerated approvals represent the same degree of therapeutic advance [2]. Other work has shown that the pivotal evidence supporting FDA approvals varies substantially in number, design, blinding, comparator use, and endpoint selection [4]. Our study connects these two literatures. It evaluates expedited pathways not only as individual designations, but as product-level portfolios, and it links those portfolios to the architecture of pivotal evidence.

The Regulatory Bundling Index was developed to summarize this portfolio effect. It should not be interpreted as implying that all designations act through the same mechanism. They do not. Priority Review directly affects review timing. Fast Track and Breakthrough Therapy designation may influence development interaction and regulatory communication. Accelerated Approval depends heavily on whether a surrogate or intermediate clinical endpoint is considered reasonably likely to predict clinical benefit. Orphan designation reflects disease prevalence and incentives, not review acceleration alone. RBI is therefore best understood as a measure of pathway-portfolio intensity, not as a substitute for the individual regulatory meaning of each program.

This distinction is important because expedited pathways are designed to facilitate development and review for serious conditions with unmet medical need, but they do not remove the need for credible evidence [9]. The accelerated approval literature makes this especially clear. Accelerated Approval can permit earlier approval based on surrogate or intermediate endpoints, but confirmatory evidence is still required to verify clinical benefit [6]. Recent oncology analyses further show why this issue remains important: many cancer indications receiving Accelerated Approval do not demonstrate overall-survival or quality-of-life benefit within five years, even though some are converted to regular approval [7]. These observations do not weaken the rationale for expedited pathways. They show that acceleration and evidentiary uncertainty must be considered together.

Our oncology–neurology contrast fits this broader literature. Oncology products in this cohort were more concentrated in high-RBI portfolios and had shorter review times. Neurology products were supported by more randomized, masked, and late-phase pivotal evidence. Enrollment did not differ significantly between areas, which is revealing. The difference was not merely about trial size. It was about trial structure. Neurology evidence was more controlled and more conservative. Oncology evidence was more often compatible with open-label or single-group designs, likely reflecting the role of biomarker-defined populations, response-based endpoints, rare molecular subgroups, and accepted oncology regulatory precedents.

The Scientific Reports decadal review of infectious-disease approvals provides a useful cross-therapeutic comparison [8]. That study found that infectious-disease products moved faster than non-infectious products and were more likely to receive Priority Review and Fast Track, but less likely to receive Accelerated Approval. The authors interpreted this pattern as regulatory alignment with disease context rather than simple underuse of a pathway. Our findings support a similar interpretation. Oncology, neurology, and infectious diseases do not use expedited programs in identical ways because their evidentiary problems are different. Pathway use appears to depend on the maturity of endpoints, the disease course, the feasibility of shorter trials, and the regulatory credibility of available evidence.

This point helps answer a likely reviewer concern: the lower RBI observed in neurology should not automatically be interpreted as regulatory neglect or inefficient strategy. Many neurological disorders rely on functional outcomes, longer follow-up, heterogeneous populations, slower disease progression, and endpoints with greater measurement variability. These features can make oncology-like acceleration difficult to transfer directly. A pathway portfolio that is highly effective in one therapeutic area may have a smaller effect in another if the endpoint structure is less mature or the evidence package requires more controlled testing.

The counterfactual analysis is consistent with this interpretation. Raising neurology products to the oncology median RBI was associated with shorter predicted review time, but the reduction was moderate. The primary scenario reduced median predicted regulatory approval time by 37.8 days, while the higher-intensity RBI 4 scenario reduced it by 71.5 days. These estimates are meaningful, but they do not imply that neurology could simply adopt oncology-like designation patterns and reproduce oncology-like timelines. The more defensible interpretation is narrower: pathway intensification may compress review time when the evidence package is ready for that form of regulatory facilitation.

The economic translation should also be read cautiously. Under the primary counterfactual scenario, the modeled reduction in neurology review time corresponded to a median RAT-related opportunity-cost reduction of $37.8 million. Under the RBI 4 scenario, the median reduction was $71.5 million. These values make the timing differences interpretable, but they are not realized savings. They are scenario-based translations. Clinical development time remained the dominant component of total pre-launch opportunity cost, which means that review-time compression alone is unlikely to solve the broader efficiency problem.

Overall, this study supports a pathway-evidence alignment framework. Expedited pathways matter, but their value depends on scientific context. A high-intensity pathway portfolio may shorten review when the endpoint, disease biology, and trial design are compatible with accelerated regulatory decision-making. Where endpoint validity is uncertain or clinical outcomes require longer observation, the same portfolio may have a smaller effect. This is the main contribution of the present analysis: it moves the discussion from whether expedited pathways are “used” toward whether they are aligned with the evidence package they are intended to accelerate.

### Policy implications

These findings support a policy framework centered on pathway-evidence alignment rather than simple expansion of expedited-designation use. For neurology, the goal should not be to copy oncology-style regulatory portfolios, because the scientific foundations of approval often differ across these fields. A more practical approach is to identify when neurology products have endpoints, biomarkers, natural-history evidence, or disease subgroups mature enough to justify earlier and more intensive regulatory engagement. In that setting, expedited programs may be used more strategically, especially when trial design and endpoint credibility are already aligned with the intended pathway. This interpretation is consistent with recent infectious-disease evidence showing that expedited programs can operate differently across therapeutic areas, with pathway use shaped by endpoint structure and disease context rather than by a universal acceleration model [8]. It also fits emerging work on operational complexity, which suggests that trial burden is a structural feature of development programs and not merely a secondary analytic covariate [10,11]. Against this background, regulators and sponsors may benefit from treating endpoint qualification, biomarker validation, adaptive-design planning, and early evidence-generation strategy as part of regulatory pathway planning itself. The broader implication is that regulatory speed should not be evaluated only by counting designations. A product with fewer designations but a mature evidence package may be more review-ready than a product with multiple designations but unresolved uncertainty. For neurology and other non-oncology fields, the highest-value reforms may therefore occur before submission: clearer evidentiary expectations, earlier FDA interaction, stronger surrogate or intermediate endpoint validation, and development strategies that make expedited pathways scientifically usable rather than merely administratively available.

### Limitations

This study has limitations. The analysis was observational, so associations between RBI, pivotal evidence architecture, and regulatory approval time should not be interpreted as causal effects. RBI is also a summary measure; it captures pathway-portfolio intensity but not the timing, qualitative value, or mechanism of each designation. The pivotal-trial dataset was intentionally conservative and FDA-source anchored, which reduced over-inclusive linkage but may have excluded supportive studies, non-NCT evidence, or trials not clearly identified in FDA materials. Evidence complexity was measured using structured features such as randomization, masking, phase, intervention model, enrollment, and link structure, but these variables cannot fully capture disease-specific challenges such as endpoint variability, placebo response, slow progression, biomarker uncertainty, crossover, or post-approval obligations. Because the cohort included only products that ultimately received FDA approval, the analysis is conditional on approval and cannot estimate progression from early development or regulatory designation to approval, abandonment, withdrawal, or non-approval. The counterfactual and economic analyses were exploratory and scenario-based; they cannot prove that assigning additional designations would shorten review time in practice, and the cost translation depends on simplified assumptions. Finally, the study was restricted to FDA oncology and neurology approvals from 2015 to 2024, so external validation in other therapeutic areas, regulatory periods, and non-US systems is needed.

## Conclusion

In this curated analysis of 195 FDA oncology and neurology approvals from 2015 to 2024, differences in regulatory approval time were best explained by considering both pathway configuration and pivotal evidence architecture. Oncology products had higher expedited-pathway portfolio intensity and shorter review intervals. Neurology products had lower pathway intensity and longer review intervals, but also more randomized, masked, and late-phase pivotal evidence. These findings do not support a simple conclusion that more expedited designations alone will make approvals faster. Instead, they suggest that pathway intensity is most useful when it fits the disease context, endpoint maturity, and trial design. The practical message is one of alignment: for neurology and other non-oncology fields, future gains may depend less on copying oncology and more on building the evidentiary conditions that make expedited pathways scientifically appropriate.

## Supporting information

S1 File Supplementary methods, reporting checklists, tables, figures, statistical outputs, robustness analyses, and reproducibility materials supporting the comparative analysis of FDA oncology and neurology approvals. The file includes S1–S10 Tables and S1–S6 Figures.
(DOCX)

## References

[1] U.S. Food and Drug Administration. Expedited programs for serious conditions—drugs and biologics. U.S. Food and Drug Administration; 2014.

[2] Kesselheim AS, Wang B, Franklin JM, Darrow JJ. Trends in utilization of FDA expedited drug development and approval programs, 1987-2014: cohort study. BMJ. 2015;:h4633. doi: 10.1136/bmj.h4633 PMC4580726 26400751

[3] Monge AN, Sigelman DW, Temple RJ, Chahal HS. Use of US Food and Drug Administration expedited drug development and review programs by orphan and nonorphan novel drugs approved from 2008 to 2021. JAMA Netw Open. 2022;5(11):e2239336. doi: 10.1001/jamanetworkopen.2022.39336 36318210 PMC9627417

[4] Downing NS, et al. Clinical trial evidence supporting FDA approval of novel therapeutic agents, 2005-2012. JAMA. 2014;311(4). 10.1001/jama.2013.282034 PMC4144867 24449315

[5] Zhang AD, et al. Assessment of clinical trials supporting US Food and Drug Administration approval of novel therapeutic agents, 1995-2017. JAMA Network Open. 2020;3(4):e203284–e203284. 10.1001/jamanetworkopen.2020.3284 PMC7175081 32315070

[6] Naci H, Smalley KR, Kesselheim AS. Characteristics of preapproval and postapproval studies for drugs granted accelerated approval by the US Food and Drug Administration. JAMA. 2017;318(7):626–36. 28810023 10.1001/jama.2017.9415 PMC5817559

[7] Liu ITT, Kesselheim AS, Cliff ERS. Clinical benefit and regulatory outcomes of cancer drugs receiving accelerated approval. JAMA. 2024;331(17):1471–9. doi: 10.1001/jama.2024.2396 38583175 PMC11000139

[8] Tam NT, Sayed AM, Dadam MN, Elsheikh R, Aziz JMA, Gad AG, et al. Regulatory alignment in FDA expedited pathways for infectious diseases: a decadal review with predictive modeling insights. Sci Rep. 2025;16(1):860. doi: 10.1038/s41598-025-30452-0 41390829 PMC12780218

[9] FDA. Guidance for Industry Expedited Programs for Serious Conditions – Drugs and. FDA. 2014. Available from: https://www.fda.gov/media/86377/download

[10] Getz KA, Campo RA. New benchmarks characterizing growth in protocol design complexity. Ther Innov Regul Sci. 2018;52(1):22–8. doi: 10.1177/2168479017713039 29714620

[11] Florez M, Smith Z, Olah Z, Martin M, Getz K. Quantifying site burden to optimize protocol performance. Ther Innov Regul Sci. 2024;58(2):347–56. doi: 10.1007/s43441-023-00602-5 38191957

