# Supplementary material for: Expedited pathway portfolios, trial complexity, and FDA approval timelines in oncology and neurology: A comparative analysis
Source: PLoS One. 2026 Aug 27;21(8):e0355495. doi: 10.1371/journal.pone.0355495 (PMC13521384; doi:10.1371/journal.pone.0355495)
Supplement: Supplementary file 1 [file pone.0355495.s001.docx]

**Expedited pathway portfolios, trial complexity, and FDA approval timelines in oncology and neurology: a comparative analysis**

*Ahmed M. Sayed*^1,2#^, *Nada M. Ezzelarab^3^*, *Nguyen Tien Huy*^2,4, 5,6^

**Affiliations**

*^1^* *Department of Chemistry, College of Science, Purdue University, West Lafayette, IN 47907, USA*

*^2^ Online Research Club. Nagasaki, Japan*

*^3^ Department of Biology, School of Sciences and Engineering, The American University in Cairo, Cairo, 11835, Egypt*

*^4^ School of Tropical Medicine and Global Health, Nagasaki University, Nagasaki 852-8523, Japan*

*^5^ Institute of Research and Development, Duy Tan University, Da Nang, Vietnam*

*^6^School of Medicine and Pharmacy, Duy Tan University, Da Nang, Vietnam*

**Corresponding author: AMS (E-Mail:* [soliman1@purdue.edu](mailto:soliman1@purdue.edu)*)*

**Emails and ORCID**

Ahmed M. Sayed: [soliman1@purdue.edu](mailto:soliman1@purdue.edu); (ORCID: 0000-0003-0244-3457)

Nada Ezzelarab: [nadaezzelarab@aucegypt.edu](mailto:nadaezzelarab@aucegypt.edu) (ORCID: 0000-0001-7992-7260)

Nguyen Tien Huy: [nguyentienhuy4@duytan.edu.vn](mailto:nguyentienhuy4@duytan.edu.vn); (ORCID: 0000-0002-9543-9440)

**Supplementary Information**

**Supplementary Methods**

## **S1.1 Reporting Standards**

The observational comparative cohort component was reported using the STROBE recommendations for observational studies. Because the manuscript also includes a supplementary predictive sensitivity analysis and an exploratory scenario-based economic translation, relevant TRIPOD and CHEERS items were mapped where applicable. These components were not designed as a primary prediction-model development study or a formal cost-effectiveness analysis. Completed STROBE reporting mapping, TRIPOD applicability mapping, and CHEERS applicability mapping are provided as Checklists 1–3.

**S1.2 Study design and data sources**

We conducted a retrospective comparative cohort study of original U.S. Food and Drug Administration approvals in oncology and neurology from January 1, 2015, through December 31, 2024. Regulatory data were obtained from publicly available FDA sources, including Drugs@FDA, FDA approval letters, product labels, and FDA review documents. Extracted product-level variables included product name, therapeutic area, application type, approval date, NDA/BLA submission date when available, and regulatory designation status. The regulatory designations abstracted were Fast Track, Breakthrough Therapy designation, Priority Review, Accelerated Approval, and Orphan designation. Designation status was coded from FDA approval materials and product-specific regulatory documents. When discrepancies were encountered across sources, the most product-specific FDA documentation was prioritized.

**S1.3 Eligibility criteria and product-level cohort construction**

**Eligible records were original FDA approvals assigned to oncology or neurology at first approval. Products were included if they were approved as new molecular entities under a New Drug Application or as original Biologics License Applications. Supplemental approvals, generic products, biosimilars, reformulations, route or dosage-form changes without a new active entity, and products regulated solely as devices or diagnostics were excluded. The final product-level cohort included 195 original FDA approvals: 136 oncology products and 59 neurology products. Product-level analyses used all 195 approvals, including products without a printed valid NCT identifier when the regulatory approval information was otherwise source verified.**

**S1.4 Pivotal-trial identification and exact NCT enrichment**

**Pivotal or registrational evidence supporting first FDA approval was identified from FDA labels and review documents. The goal was to capture trials that supported the original approval decision, not every trial associated with a product. Trials explicitly described by FDA as pivotal, registrational, adequate and well controlled, or primary evidence supporting approval were prioritized. NCT identifiers were included only when they were printed in FDA source materials and could be manually verified. Products with FDA-described pivotal evidence but no printed valid NCT identifier were retained in product-level regulatory analyses but excluded from exact NCT-enriched evidence-architecture analyses. Curated NCT identifiers were enriched through exact matching to AACT, the relational database derived from ClinicalTrials.gov. No fuzzy matching, product-name-only matching, or broad ClinicalTrials.gov search matching was used. The final curated evidence dataset contained 221 product–NCT pivotal links, representing 220 distinct NCT identifiers, and all 221 links were enriched by exact AACT matching.**

**S1.5 Units of analysis**

**Two analytic levels were used.**

**First, product-level analyses used the regulatory application as the unit of analysis and included all 195 approvals. These analyses evaluated regulatory designations, Regulatory Bundling Index, approval timelines, and the number of curated pivotal NCT links per product.**

**Second, product–NCT link-level analyses used each curated pivotal product–NCT link as the unit of analysis and were restricted to the 221 exact NCT-enriched pivotal links. These analyses evaluated pivotal evidence architecture, including allocation, masking, phase, intervention model, enrollment, and exploratory design complexity.**

**S1.6 Regulatory timeline definitions**

**Regulatory approval time was defined as the number of calendar days from NDA/BLA submission to FDA approval. Clinical development time was defined as the interval from the earliest stable first-in-human or first clinical-development anchor date to NDA/BLA submission. Total time to approval was defined as clinical development time plus regulatory approval time. Date anchors were selected using prespecified precedence rules. Products without a stable anchor date for a specific interval were excluded only from analyses requiring that interval. Regulatory approval time was reported in days, whereas clinical development time and total time to approval were reported in years. Missing timeline values were not imputed for primary analyses.**

**S1.7 Regulatory Bundling Index**

**The primary regulatory exposure was expedited pathway portfolio intensity, summarized using the Regulatory Bundling Index. RBI was calculated as the sum of five binary indicators: Fast Track, Breakthrough Therapy designation, Priority Review, Accelerated Approval, and Orphan designation. Scores ranged from 0 to 5, with higher values indicating a greater number of expedited or orphan designations.**

**Orphan designation was included because it reflects an important regulatory-development context and frequently co-occurs with FDA expedited programs, although it is not itself one of the four FDA expedited programs for serious conditions. RBI should therefore be interpreted as a pathway-portfolio intensity measure, not as evidence that all included designations act through the same regulatory mechanism.**

**S1.8 Evidence-architecture variables and exploratory design-complexity score**

**Evidence-architecture variables were extracted from exact AACT-enriched NCT records. Allocation was classified as randomized or not randomized. Masking was classified as masked or not masked. Phase 3 and phase 2/3 studies were classified as late phase. Intervention model was used to identify single-group designs. Enrollment was analyzed using the AACT-reported enrollment value.**

**An exploratory design-complexity score was used to summarize structured evidence-architecture features. The score incorporated randomized allocation, masking, late-phase status, non-single-group design, and enrollment scale. The enrollment component was scaled as min(log10(enrollment + 1) / 4, 1) to reduce the influence of very large trials. The total score was calculated as the sum of randomized allocation, masking, late-phase status, non-single-group design, and the scaled enrollment component, giving a possible range from 0 to 5. This score was used descriptively to summarize evidence architecture and was not treated as a validated clinical-development burden instrument.**

**S1.9 Descriptive and comparative statistical analyses**

**Continuous and ordinal variables were summarized as medians and interquartile ranges. Categorical variables were summarized as counts, denominators, and percentages. Oncology and neurology were compared using Wilcoxon rank-sum tests for continuous or ordinal variables and Fisher exact tests for categorical variables. For major binary evidence-architecture features, absolute percentage-point differences were calculated as neurology minus oncology and reported with 95% confidence intervals. All P values were two-sided. Exact P values were reported when P ≥ 0.001, and smaller values were reported as P < 0.001. The primary analyses were descriptive and comparative. They were designed to quantify between-area differences in pathway intensity, approval timelines, and pivotal evidence architecture. No causal effect of therapeutic area or designation status was assumed.**

**S1.10 Joint pathway-evidence mapping**

**To explore how pathway intensity and evidence architecture jointly structured review time, product-level RBI and product-level evidence complexity were grouped into prespecified strata. RBI was grouped as RBI 0–1, RBI 2, and RBI 3–5. Evidence complexity was grouped into lower, middle, and higher complexity bands. Median regulatory approval time was summarized within populated therapeutic-area strata. Cell counts were reported for each stratum, and low-count cells were interpreted cautiously. These analyses were descriptive and were intended to visualize the pathway-evidence timing landscape rather than to estimate causal effects.**

**S1.11 Exploratory counterfactual simulation**

**Exploratory counterfactual analyses estimated how predicted neurology regulatory approval time might change under higher pathway-intensity scenarios. A product-level log-linear model was fit using log-transformed regulatory approval time as the outcome. Predictors included therapeutic area, Regulatory Bundling Index, product-level evidence complexity, log-transformed enrollment, and the number of curated pivotal NCT links per product. The primary counterfactual scenario raised neurology products with RBI below the oncology median to the oncology median RBI while preserving therapeutic area, evidence-complexity measures, enrollment summaries, and curated NCT-link structure. A second scenario raised eligible neurology products to the oncology upper-quartile RBI. Predicted regulatory approval times were back-transformed to days. Uncertainty was summarized using bootstrap resampling with 2,000 iterations. These analyses were exploratory and scenario-based. They should not be interpreted as causal evidence that assigning additional designations would necessarily shorten FDA review time in practice.**

**S1.12 Economic translation**

**An exploratory economic translation was conducted to express modeled review-time differences in opportunity-cost terms. The base-case assumptions assigned $1.0 million to each regulatory approval day and $0.5 million to each clinical-development day. Clinical development time was held constant in counterfactual scenarios so that the translation isolated modeled changes in regulatory approval time. Economic outputs were reported as scenario-based translations of predicted timing differences, not as realized savings. No quality-adjusted life-years, payer-perspective utility outcomes, or incremental cost-effectiveness ratios were modeled.**

**S1.13 Robustness analyses**

**Robustness analyses repeated the regulatory approval time comparison in the full product-level cohort, in the subset of products with valid curated NCT identifiers, after excluding the top 1% of regulatory approval time values, and after winsorizing regulatory approval time values. These analyses assessed whether the primary regulatory approval time difference was sensitive to source coverage or extreme values.**

**S1.14 Supplementary predictive sensitivity analysis**

**A supplementary gradient-boosted predictive model was fit only as a predictive sensitivity analysis. It was not used for primary inference. The model used product-level pathway-intensity, evidence-architecture, enrollment, source-coverage, and therapeutic-area variables to predict log-transformed regulatory approval time. Out-of-fold predictions were used to evaluate predictive performance. Model performance was summarized using R-squared on the log scale and error metrics after back-transformation to days. Feature importance was reported descriptively.**

**S1.15 Software, code, and reproducibility**

All analyses were performed in R using a reproducible scripted workflow. The workflow generated analysis-ready datasets, product-level and product–NCT link-level tables, figure-source data, individual supplementary figures, composite manuscript figures, robustness outputs, exploratory counterfactual outputs, economic translation outputs, and a clean supplementary ZIP package.

The main workflow used packages for data processing, visualization, tabulation, bootstrapping, and model summaries, including readr, dplyr, tidyr, stringr, ggplot2, scales, janitor, lubridate, openxlsx, tibble, purrr, forcats, broom, and patchwork. The supplementary predictive sensitivity analysis used xgboost when available. Random seeds were fixed for stochastic procedures. The complete analysis code, processed datasets, output tables, generated figures, quality-control files, and file manifest are publicly available in the Zenodo repository (DOI: 10.5281/zenodo.21606436).

**SUPPLEMENTARY CHECKLISTS**

**Checklist 1: STROBE checklist**

| Item No. | Recommendation | Page / section | Relevant text from manuscript |
| --- | --- | --- | --- |
| Title and abstract | 1(a) Indicate the study’s design with a commonly used term in the title or the abstract | Title; Abstract | The title identifies the work as “a comparative analysis.” The Abstract states that the study was a retrospective comparative cohort study of FDA original approvals in oncology and neurology from 2015 to 2024. |
| Title and abstract | 1(b) Provide in the abstract an informative and balanced summary of what was done and what was found | Abstract | The Abstract summarizes the objective, cohort size, therapeutic-area groups, regulatory data sources, exact NCT enrichment, Regulatory Bundling Index, timeline outcomes, pivotal evidence architecture, principal results, and conclusion. |
| Introduction | 2 Explain the scientific background and rationale | Introduction | The Introduction describes FDA expedited programs, differences between oncology and neurology evidence packages, and the rationale for evaluating pathway intensity together with pivotal evidence architecture. |
| Introduction | 3 State specific objectives | Introduction | The Introduction states that the study evaluates whether review-time differences reflect regulatory configuration alone, evidentiary burden alone, or alignment between pathway intensity and pivotal evidence architecture. |
| Methods | 4 Present key elements of study design early | Methods: Study design and reporting | The Methods state that this was a retrospective comparative cohort study of original FDA oncology and neurology approvals from January 1, 2015, through December 31, 2024. |
| Methods | 5 Describe setting, locations, dates | Methods: Study design and reporting; Data sources and regulatory curation | The setting was U.S. FDA original approvals from 2015 to 2024. Product-level regulatory data were obtained from Drugs@FDA, FDA approval letters, product labels, and FDA review documents. The regulatory database was accessed on August 1, 2025. |
| Methods | 6(a) Eligibility criteria, sources, and methods of selection | Methods: Eligibility criteria; Data sources and regulatory curation | Eligible products were original FDA approvals assigned to oncology or neurology at first approval, including new molecular entities under New Drug Applications and original Biologics License Applications. Supplemental approvals, generics, biosimilars, reformulations, route or dosage-form changes without a new active entity, and products regulated solely as devices or diagnostics were excluded. |
| Methods | 6(b) For matched studies, give matching criteria | Not applicable | Not applicable. This was not a matched cohort study. No propensity-score matching, treatment matching, or matched sampling design was used in the revised analysis. |
| Methods | 7 Define all outcomes, exposures, predictors, potential confounders, and effect modifiers | Methods: Outcomes; Exposures and evidence-architecture variables | Outcomes included regulatory approval time, clinical development time, total time to approval, and pivotal evidence architecture. The primary regulatory exposure was Regulatory Bundling Index, calculated from Fast Track, Breakthrough Therapy designation, Priority Review, Accelerated Approval, and Orphan designation. Evidence-architecture variables included allocation, masking, late-phase status, intervention model, enrollment, and exploratory design complexity. |
| Methods | 8 Data sources and measurement details | Methods: Data sources and regulatory curation; Pivotal-trial identification and exact NCT enrichment | Product-level regulatory data were obtained from FDA approval materials. Pivotal or registrational evidence was identified from FDA labels and review documents. NCT identifiers were included only when printed in FDA source materials and manually verified. Curated NCT identifiers were enriched through exact matching to AACT. No fuzzy matching, product-name-only matching, or broad ClinicalTrials.gov search matching was used. |
| Methods | 9 Address efforts to mitigate bias | Methods: Pivotal-trial identification and exact NCT enrichment; Statistical analysis; Limitations | Bias from over-inclusive trial linkage was reduced by using FDA-source-anchored pivotal-trial curation and exact NCT enrichment only. Products without printed valid NCT identifiers were retained in product-level analyses but excluded from exact NCT-enriched evidence-architecture analyses. The manuscript states that the analysis is observational and should not be interpreted causally. |
| Methods | 10 Explain how study size was determined | Methods: Data sources and regulatory curation; Results: Cohort construction | The study included all eligible original FDA oncology and neurology approvals from 2015 to 2024 that met the prespecified criteria. The final product-level cohort included 195 approvals: 136 oncology products and 59 neurology products. |
| Methods | 11 Explain how quantitative variables were handled | Methods: Outcomes; Exposures and evidence-architecture variables; Statistical analysis | Regulatory approval time was reported in days. Clinical development time and total time to approval were reported in years. Enrollment was analyzed using the AACT-reported value and log-transformed where used in model-based analyses. The exploratory design-complexity score summarized structured evidence-architecture features and was used descriptively. |
| Methods | 12(a) Describe all statistical methods | Methods: Statistical analysis; Joint pathway-evidence, counterfactual, and sensitivity analyses | Continuous and ordinal variables were summarized as medians and interquartile ranges. Categorical variables were summarized as counts, denominators, and percentages. Oncology and neurology were compared using Wilcoxon rank-sum tests for continuous or ordinal variables and Fisher exact tests for categorical variables. Percentage-point differences with 95% confidence intervals were reported for major binary evidence-architecture features. |
| Methods | 12(b) Describe subgroup analyses and interactions | Methods: Joint pathway-evidence, counterfactual, and sensitivity analyses | Descriptive pathway-evidence mapping was performed by grouping product-level Regulatory Bundling Index and product-level evidence complexity into prespecified strata and summarizing median regulatory approval time within populated therapeutic-area strata. No formal causal interaction model was used in the revised primary analysis. |
| Methods | 12(c) Explain how missing data were addressed | Methods: Statistical analysis | Missing data were handled using complete-case analysis for the variable or interval being evaluated. Products without valid curated NCT identifiers were retained in product-level regulatory analyses but excluded from exact NCT-enriched evidence-architecture analyses. |
| Methods | 12(d) Explain analytical methods accounting for sampling strategy | Not applicable | Not applicable. This was not a survey sample and did not require survey-weighted estimation. Product-level analyses used FDA approvals as the unit of analysis, and product–NCT link-level analyses used exact NCT-enriched pivotal links as the unit of analysis. |
| Methods | 12(e) Describe sensitivity analyses | Methods: Joint pathway-evidence, counterfactual, and sensitivity analyses | Robustness analyses repeated the regulatory approval time comparison in the full cohort, in the valid-NCT subset, after excluding the top 1% of regulatory approval time values, and after winsorizing regulatory approval time values. A supplementary gradient-boosted model was fit only as a predictive sensitivity analysis and was not used for primary inference. |
| Results | 13(a) Report numbers at each stage of study | Results: Cohort construction | The final curated cohort included 195 original FDA approvals: 136 oncology products and 59 neurology products. Of these, 174 products had at least one valid curated NCT identifier, and the exact NCT-enriched evidence dataset included 221 product–NCT pivotal links representing 220 distinct NCT identifiers. |
| Results | 13(b) Give reasons for non-participation at each stage | Not applicable | Not applicable. This was a study of public regulatory records, not a participant-recruitment study. Exclusions were based on prespecified regulatory eligibility criteria. |
| Results | 13(c) Consider use of a flow diagram | Results; S1 Fig | The denominator and linkage structure is summarized in the Results and shown in S1 Fig, including the product-level cohort, valid curated NCT coverage, product–NCT pivotal links, distinct NCT identifiers, and exact AACT-matched links. |
| Results | 14(a) Give characteristics of study participants | Results; Tables 1–2 | Table 1 reports product-level regulatory characteristics and approval timelines by therapeutic area. Table 2 reports curated pivotal evidence architecture by therapeutic area. |
| Results | 14(b) Indicate number of participants with missing data for each variable | Results; Tables 1–2; Supporting Information | The manuscript reports variable-specific denominators where applicable. Products without valid curated NCT identifiers were retained in product-level analyses but excluded from exact NCT-enriched evidence-architecture analyses. |
| Results | 14(c) Summarize follow-up time | Not applicable | Not applicable. This was not a longitudinal participant follow-up study. Regulatory and development intervals were calculated from public date anchors. |
| Results | 15 Report outcome data | Results; Tables 1–3 | The Results report regulatory approval time, clinical development time, total time to approval, pathway intensity, pivotal evidence architecture, counterfactual predicted days saved, and economic translation outputs. |
| Results | 16(a) Report unadjusted and adjusted estimates and their precision | Results; Tables 1–3; Supporting Information | The primary analyses report descriptive and comparative estimates by therapeutic area, including medians, interquartile ranges, counts, percentages, p values, and percentage-point differences with 95% confidence intervals. The counterfactual model outputs and bootstrap intervals are reported separately as exploratory scenario-based analyses. |
| Results | 16(b) Report category boundaries when continuous variables are categorized | Methods: Joint pathway-evidence, counterfactual, and sensitivity analyses; Results: Joint pathway-evidence map | Regulatory Bundling Index was grouped as RBI 0–1, RBI 2, and RBI 3–5 for pathway-evidence mapping. Evidence complexity was grouped into lower, middle, and higher complexity bands. |
| Results | 16(c) Translate estimates into absolute risk if relevant | Not applicable | Not applicable. The study did not estimate risk ratios, odds ratios, hazard ratios, or absolute clinical risks. Timing differences were reported directly in days or years, and exploratory economic translation was reported separately. |
| Results | 17 Other analyses | Results: Joint pathway-evidence map; Counterfactual analyses; Robustness and predictive sensitivity analyses | Other analyses included descriptive pathway-evidence mapping, exploratory counterfactual simulation, economic translation, robustness analyses, and a supplementary predictive sensitivity analysis. |
| Discussion | 18 Summarize key results | Discussion | The Discussion summarizes that oncology products had higher pathway intensity and shorter regulatory review time, while neurology products had lower pathway intensity, longer review intervals, and more conservative pivotal evidence architecture. |
| Discussion | 19 Discuss limitations | Limitations | The Limitations section states that the analysis is observational, conditional on FDA approval, restricted to FDA oncology and neurology approvals from 2015 to 2024, and that counterfactual and economic outputs are exploratory and scenario-based rather than causal estimates or realized savings. |
| Discussion | 20 Interpretation considering objectives, limitations, multiplicity of analyses, and similar studies | Discussion; Policy Implications | The Discussion interprets the findings as supporting a pathway-evidence alignment framework rather than a simple model in which more expedited designations alone explain faster approval. |
| Discussion | 21 Discuss generalizability | Limitations; Conclusion | The manuscript states that external validation in other therapeutic areas, regulatory periods, and non-U.S. systems is needed. |
| Other information | 22 Give source of funding and role of funders | Declarations | “This research received no external funding.” |

**Checklist 2. TRIPOD Applicability Mapping for Supplementary Predictive Sensitivity Analysis**

| Section / Topic | Item | Checklist Item | Page / section | Relevant text from manuscript |
| --- | --- | --- | --- | --- |
| Title and abstract | 1 | Identify the study as developing and/or validating a multivariable prediction model, the target population, and the outcome to be predicted. | Title; Abstract | Partially applicable. The manuscript is not presented as a prediction-model development or validation study. The title and Abstract identify the work as a comparative analysis of FDA oncology and neurology approvals. The supplementary gradient-boosted model was used only as a predictive sensitivity analysis for regulatory approval time and was not used for primary inference. |
| Title and abstract | 2 | Provide a summary of objectives, study design, setting, participants, sample size, predictors, outcome, statistical analysis, results, and conclusions. | Abstract | The Abstract summarizes the objective, retrospective comparative cohort design, setting, sample size of 195 original FDA approvals, therapeutic-area groups, regulatory variables, timeline outcomes, pivotal evidence architecture, main results, and conclusions. The predictive sensitivity analysis is not emphasized in the Abstract because it was supplementary and not part of the primary inference. |
| Introduction | 3a | Explain the medical context and rationale, including references to existing models. | Introduction | The Introduction describes the regulatory context of FDA expedited programs, differences between oncology and neurology evidence packages, and the rationale for studying pathway intensity together with pivotal evidence architecture. The manuscript does not claim that a prior validated prediction model exists for this setting. |
| Introduction | 3b | Specify the objectives, including whether the study is for development or validation of the model or both. | Introduction; Methods | The primary objective was not prediction-model development or validation. The objective was to compare FDA oncology and neurology approvals with respect to pathway intensity, approval timelines, and pivotal evidence architecture. A supplementary gradient-boosted model was fit only as a predictive sensitivity analysis. |
| Methods | 4a | Describe study design or source of data, separately for development and validation data sets. | Methods: Study design and reporting; Statistical analysis | The study was a retrospective comparative cohort study using publicly available FDA regulatory sources and exact NCT enrichment through AACT. No separate model-development and external-validation datasets were defined because the predictive analysis was supplementary and exploratory. |
| Methods | 4b | Specify key study dates. | Methods: Study design and reporting | The cohort included original FDA oncology and neurology approvals from January 1, 2015, through December 31, 2024. The regulatory database was accessed on August 1, 2025. |
| Methods | 5a | Specify key elements of the study setting. | Methods: Data sources and regulatory curation | The setting was U.S. FDA original drug and biologic approvals. Product-level regulatory data were obtained from Drugs@FDA, FDA approval letters, product labels, and FDA review documents. Pivotal-trial metadata were enriched through exact NCT matching to AACT. |
| Methods | 5b | Describe eligibility criteria for participants. | Methods: Eligibility criteria | Eligible records were original FDA approvals assigned to oncology or neurology at first approval, including new molecular entities under New Drug Applications and original Biologics License Applications. Supplemental approvals, generics, biosimilars, reformulations, route or dosage-form changes without a new active entity, and products regulated solely as devices or diagnostics were excluded. |
| Methods | 5c | Give details of treatments received, if relevant. | Not applicable | Not applicable. The study analyzed FDA regulatory approvals and product-level evidence packages, not patient-level treatment assignment or individual participant outcomes. |
| Methods | 6a | Clearly define the outcome predicted. | Methods: Outcomes; Supplementary predictive sensitivity analysis | The predicted outcome in the supplementary model was regulatory approval time, defined as the number of calendar days from NDA/BLA submission to FDA approval. For model fitting, regulatory approval time was log-transformed. |
| Methods | 6b | Report any actions to blind assessment of the outcome. | Not applicable | Not applicable. Regulatory approval time was derived from public FDA regulatory dates, and no subjective blinded outcome adjudication was required. |
| Methods | 7a | Define all predictors, including how and when measured. | Methods: Exposures and evidence-architecture variables; Supplementary predictive sensitivity analysis | Candidate predictors for the supplementary predictive sensitivity analysis included product-level pathway-intensity variables, Regulatory Bundling Index, therapeutic area, evidence-architecture variables, enrollment, source-coverage variables, and curated pivotal NCT-link count. Endpoint-tier variables were not used in the revised analytic framework. |
| Methods | 7b | Report any actions to blind assessment of predictors. | Not applicable | Not applicable. Predictors were extracted from public FDA sources and exact AACT-enriched NCT records using a reproducible curation workflow. |
| Methods | 8 | Explain how the study size was arrived at. | Methods: Data sources and regulatory curation; Results: Cohort construction | The study included all eligible original FDA oncology and neurology approvals from 2015 to 2024 that met the prespecified criteria. The final product-level cohort included 195 approvals: 136 oncology products and 59 neurology products. |
| Methods | 9 | Describe how missing data were handled. | Methods: Statistical analysis | Missing data were handled using complete-case analysis for the variable or interval being evaluated. Products without valid curated NCT identifiers were retained in product-level analyses but excluded from exact NCT-enriched evidence-architecture analyses. |
| Methods | 10a | Describe how predictors were handled in analyses. | Methods: Exposures and evidence-architecture variables; Joint pathway-evidence, counterfactual, and sensitivity analyses | Regulatory Bundling Index was analyzed as a pathway-portfolio intensity score. Evidence-architecture variables were coded from exact AACT-enriched NCT records. Enrollment was analyzed using the AACT-reported value and log-transformed where used in model-based analyses. |
| Methods | 10b | Specify type of model, model-building procedures, and internal validation. | Methods: Joint pathway-evidence, counterfactual, and sensitivity analyses; Supplementary Methods | A supplementary gradient-boosted model was fit only as a predictive sensitivity analysis. The outcome was log-transformed regulatory approval time. Out-of-fold prediction was used to summarize predictive performance. The model was not used for primary inference. |
| Methods | 10d | Specify all measures used to assess model performance. | Results: Robustness and predictive sensitivity analyses; Supporting Information | Predictive performance was summarized using R-squared on the log-transformed regulatory approval time scale and error metrics after back-transformation to days, including RMSE, MAE, and median absolute error. |
| Methods | 11 | Provide details on risk group creation, if done. | Not applicable | Not applicable. No clinical risk groups or prediction-based risk strata were created. |
| Results | 13a | Describe flow of participants and numbers with/without outcome. | Results: Cohort construction | The final product-level cohort included 195 original FDA approvals. Regulatory approval time analyses used products with complete submission and approval dates. The exact NCT-enriched pivotal evidence dataset included 221 product–NCT pivotal links. |
| Results | 13b | Describe characteristics of participants. | Results; Tables 1–2 | Table 1 reports product-level regulatory characteristics and approval timelines by therapeutic area. Table 2 reports curated pivotal evidence architecture by therapeutic area. |
| Results | 14a | Specify the number of participants and events in each analysis. | Results; Supporting Information tables | Product-level analyses used the 195 FDA approvals when relevant data were available. Product–NCT link-level evidence-architecture analyses used the 221 exact NCT-enriched pivotal links. The supplementary predictive sensitivity analysis used product-level regulatory approval time as the outcome. |
| Results | 14b | If done, report unadjusted association between each candidate predictor and outcome. | Not applicable | Not applicable as a formal prediction-model development report. The manuscript emphasizes descriptive comparisons, pathway-evidence mapping, and exploratory counterfactual analyses rather than presenting unadjusted predictor-by-predictor associations for a primary prediction model. |
| Results | 15a | Present full prediction model. | Supporting Information | Partially applicable. The supplementary gradient-boosted model was not intended for clinical or regulatory deployment, and no deployable prediction equation was generated. Model performance and feature-importance summaries are provided in the Supporting Information. |
| Results | 15b | Explain how to use the prediction model. | Not applicable | Not applicable. The model was used only as a supplementary predictive sensitivity analysis and is not proposed for individual-product decision-making or prospective regulatory prediction. |
| Results | 16 | Report performance measures for the prediction model. | Results: Robustness and predictive sensitivity analyses; Supporting Information | The supplementary gradient-boosted model showed limited out-of-fold performance, with R² = 0.029 on the log-regulatory approval time scale, RMSE = 357.5 days, MAE = 137.2 days, and median absolute error = 72.4 days after back-transformation to days. |
| Discussion | 18 | Discuss limitations of the study. | Limitations | The manuscript states that the analysis is observational, conditional on FDA approval, and that the counterfactual, economic, and predictive sensitivity analyses should not be interpreted as causal or prospectively validated prediction tools. |
| Discussion | 19b | Give overall interpretation of results. | Discussion; Conclusion | The Discussion interprets the findings as supporting a pathway-evidence alignment framework. The predictive sensitivity analysis supported the decision to emphasize conservative descriptive inference rather than model-based prediction as the primary analytic strategy. |
| Discussion | 20 | Discuss potential clinical use and implications for future research. | Discussion; Policy Implications; Limitations | The manuscript does not propose direct clinical use of a prediction model. Future work could evaluate pipeline-level survival from early development or initial designation through approval, discontinuation, withdrawal, or non-approval, and could externally validate predictive models in broader regulatory datasets. |
| Other information | 21 | Provide information about availability of supplementary resources. | Data availability; Supporting Information | The Supporting Information package includes analysis code, processed datasets, curated linkage audits, figure-source data, supplementary tables, generated figures, quality-control files, and a file manifest. |
| Other information | 22 | Give source of funding. | Declarations | “This research received no external funding.” |

**Checklist 3. CHEERS 2022 Checklist: Applicability Mapping**

| Item No. | CHEERS 2022 Recommendation | Page / section | Relevant text from manuscript |
| --- | --- | --- | --- |
| 1 | Title identifies the study as an economic evaluation, or includes terms such as “cost-effectiveness” or “economic impact” | Title page | Not applicable. This manuscript is not presented as a formal economic evaluation or cost-effectiveness analysis. The title accurately identifies the study as a comparative analysis of expedited pathway portfolios, trial complexity, and FDA approval timelines. |
| 2 | Abstract includes objectives, perspective, setting, methods, results, and conclusions | Abstract | The Abstract summarizes the study objective, setting, data sources, cohort size, regulatory variables, timeline outcomes, pivotal evidence architecture, main results, and conclusions. The economic translation is exploratory and scenario-based and is therefore reported in the main Results rather than framed as a primary economic evaluation in the Abstract. |
| 3 | Introduction states the study question and economic importance | Introduction | The Introduction frames the study around the relationship between expedited pathway use, evidentiary burden, and approval timelines across oncology and neurology. Economic implications are treated as exploratory consequences of modeled timing differences rather than as the primary study objective. |
| 4 | Target population and subgroups described | Methods: Study design and eligibility criteria | The target cohort is described as original FDA oncology and neurology approvals from 2015 to 2024. Therapeutic-area subgroups were oncology and neurology. |
| 5 | Setting and location described | Methods: Study design and data sources | The setting was U.S. FDA original approvals from 2015 to 2024. Regulatory data were obtained from Drugs@FDA, FDA approval letters, product labels, and FDA review documents, with trial metadata enriched through exact NCT matching to AACT. |
| 6 | Study perspective stated | Methods: Joint pathway-evidence, counterfactual, and sensitivity analyses; Results: Economic translation | The economic translation used an opportunity-cost framing based on prespecified per-day assumptions. It was not conducted from a payer, healthcare-system, or cost-effectiveness perspective. |
| 7 | Comparators described | Methods: Joint pathway-evidence, counterfactual, and sensitivity analyses | The descriptive comparison was between oncology and neurology approvals. The counterfactual economic translation compared predicted neurology regulatory approval time under observed pathway intensity with predicted neurology regulatory approval time after increasing pathway intensity to prespecified RBI scenarios. |
| 8 | Time horizon stated and justified | Methods: Outcomes | Regulatory approval time was measured from NDA/BLA submission to FDA approval. Clinical development time was measured from the earliest stable first-in-human or first clinical-development anchor date to NDA/BLA submission. Total time to approval was defined as clinical development time plus regulatory approval time. |
| 9 | Discount rate reported if used | — | No discount rate was applied because the analysis did not model long-term costs, health outcomes, QALYs, or cost-effectiveness over a future time horizon. The economic component used fixed per-day opportunity-cost assumptions. |
| 10 | Choice of health outcomes described | — | No health outcomes were modeled. The study outcomes were regulatory approval time, clinical development time, total time to approval, and pivotal evidence architecture. |
| 11 | Measurement of effectiveness | — | Clinical effectiveness was not estimated. The exploratory model estimated predicted regulatory approval time and predicted days saved under pathway-intensity scenarios. |
| 12 | Measurement and valuation of preference-based outcomes | — | Not applicable (no QALYs assessed). |
| 13 | Estimation of resources and costs | Methods: Joint pathway-evidence, counterfactual, and sensitivity analyses; Results: Economic translation | Opportunity-cost translation used prespecified assumptions of $1.0 million per regulatory approval time day and $0.5 million per clinical-development day. Clinical development time was held constant in counterfactual scenarios so that modeled cost differences reflected predicted changes in regulatory approval time. |
| 14 | Currency, price date, and conversion | Results: Economic translation | All monetary values were reported in U.S. dollars. The analysis used fixed per-day opportunity-cost assumptions and did not require currency conversion. |
| 15 | Choice of model | Methods: Joint pathway-evidence, counterfactual, and sensitivity analyses | Scenario-based economic translation was based on predicted regulatory approval time from a product-level log-linear model using log-transformed regulatory approval time. Predictors included therapeutic area, Regulatory Bundling Index, product-level evidence complexity, log enrollment, and curated pivotal NCT-link count. No formal cost-effectiveness model was used. |
| 16 | Assumptions stated | Methods: Joint pathway-evidence, counterfactual, and sensitivity analyses; Results: Economic translation | The base-case assumptions assigned $1.0 million to each regulatory approval day and $0.5 million to each clinical-development day. Clinical development time was held constant in counterfactual scenarios. The resulting monetary estimates were interpreted as scenario-based opportunity-cost translations, not realized savings. |
| 17 | Analytical methods described | Methods: Statistical analysis; Methods: Joint pathway-evidence, counterfactual, and sensitivity analyses | Descriptive and comparative analyses used Wilcoxon rank-sum tests for continuous or ordinal variables and Fisher exact tests for categorical variables. Counterfactual analyses used a product-level log-linear model for log-transformed regulatory approval time, back-transformation to days, and bootstrap resampling to summarize uncertainty. A supplementary gradient-boosted model was used only as a predictive sensitivity analysis. |
| 18 | Study parameters reported | Tables 1–3; Supporting Information tables | Tables report product-level regulatory characteristics, approval timelines, curated pivotal evidence architecture, design-complexity summaries, counterfactual predictions, bootstrap intervals, and economic translation outputs. |
| 19 | Incremental costs and outcomes reported | Results: Economic translation; Table 3 | Median predicted days saved under each counterfactual scenario were translated into regulatory approval time-related opportunity-cost reductions using the prespecified per-day cost assumption. |
| 20 | Characterizing uncertainty | Methods: Joint pathway-evidence, counterfactual, and sensitivity analyses; Results: Counterfactual analyses | Uncertainty in the counterfactual estimates was summarized using bootstrap intervals. Robustness analyses repeated the regulatory approval time comparison in the full cohort, in the valid-NCT subset, after excluding the top 1% of regulatory approval time values, and after winsorizing regulatory approval time values. |
| 21 | Heterogeneity reported | Results: Joint pathway-evidence map | Heterogeneity was described by therapeutic area and by pathway-evidence strata. Product-level Regulatory Bundling Index and evidence-complexity bands were used to summarize differences in median regulatory approval time across populated oncology and neurology strata. |
| 22 | Study findings, limitations, generalizability, and interpretation discussed | Discussion; Limitations | The Discussion interprets the findings as supporting a pathway-evidence alignment framework. The Limitations section states that the analysis is observational, conditional on FDA approval, and that counterfactual and economic outputs are exploratory, scenario-based translations rather than causal estimates or realized savings. |
| 23 | Source of funding | Declarations | “This research received no external funding.” |
| 24 | Conflicts of interest | Declarations | “The authors declare no relevant financial or non-financial interests.” |

**SUPPLEMENTARY TABLES**

S1 Table. Curated linkage audit and source coverage

| step | n |
| --- | --- |
| Products | 195 |
| Products with valid NCT | 174 |
| Product-NCT links | 221 |
| Distinct NCTs | 220 |
| AACT matched links | 221 |

S2 Table. Extended statistical tests summary

| comparison | p_value | p_value_fmt |
| --- | --- | --- |
| RBI by area, product level | 1.26E-06 | p < 0.001 |
| RAT by area, product level | 4.26E-09 | p < 0.001 |
| CDT by area, product level | 0.703059 | p = 0.703 |
| TTA by area, product level | 0.412778 | p = 0.413 |
| NCT links per product by area, product level | 0.013681 | p = 0.014 |
| Randomized by area, product-NCT link level | 7.29E-06 | p < 0.001 |
| Masked by area, product-NCT link level | 1.90E-26 | p < 0.001 |
| Late phase by area, product-NCT link level | 4.42E-09 | p < 0.001 |
| Single group by area, product-NCT link level | 2.07E-06 | p < 0.001 |
| Enrollment by area, product-NCT link level | 0.151738 | p = 0.152 |
| Design complexity by area, product-NCT link level | 9.09E-16 | p < 0.001 |
| Allocation group by area, product-NCT link level | 7.29E-06 | p < 0.001 |
| Masking group by area, product-NCT link level | 5.00E-05 | p < 0.001 |
| Intervention model by area, product-NCT link level | 5.00E-05 | p < 0.001 |
| Phase group by area, product-NCT link level | 5.00E-05 | p < 0.001 |

S3 Table. Pivotal evidence architecture effect-size estimates

| area | feature | n | denominator | pct | value | label | count_label |
| --- | --- | --- | --- | --- | --- | --- | --- |
| Neurology | Randomized | 77 | 79 | 97.46835443037975 | 77 (97.5%) | 97.50% | 77/79 |
| Oncology | Randomized | 107 | 142 | 75.35211267605634 | 107 (75.4%) | 75.40% | 107/142 |
| Neurology | Masked | 75 | 79 | 94.93671 | 75 (94.9%) | 94.90% | 75/79 |
| Oncology | Masked | 35 | 142 | 24.647887323943664 | 35 (24.6%) | 24.60% | 35/142 |
| Neurology | Late phase | 65 | 79 | 82.27848101265823 | 65 (82.3%) | 82.30% | 65/79 |
| Oncology | Late phase | 60 | 142 | 42.25352112676056 | 60 (42.3%) | 42.30% | 60/142 |
| Neurology | Single group | 2 | 79 | 2.5316455696202533 | 2 (2.5%) | 2.50% | 2/79 |
| Oncology | Single group | 37 | 142 | 26.056338028169012 | 37 (26.1%) | 26.10% | 37/142 |

S4 Table. Joint pathway–evidence heatmap cell values

| area | RBI_band | complexity_band | median_RAT | n |
| --- | --- | --- | --- | --- |
| Neurology | RBI 0-1 | Higher complexity | 365 | 21 |
| Neurology | RBI 0-1 | Middle complexity | 2449.5 | 2 |
| Neurology | RBI 2 | Higher complexity | 366 | 3 |
| Neurology | RBI 2 | Middle complexity | 335 | 3 |
| Neurology | RBI 3-5 | Higher complexity | 334 | 9 |
| Neurology | RBI 3-5 | Middle complexity | 326.5 | 10 |
| Neurology | RBI 3-5 | Lower complexity | 335 | 1 |
| Oncology | RBI 0-1 | Higher complexity | 320 | 6 |
| Oncology | RBI 0-1 | Middle complexity | 344 | 11 |
| Oncology | RBI 0-1 | Lower complexity | 363 | 3 |
| Oncology | RBI 2 | Higher complexity | 227.5 | 10 |
| Oncology | RBI 2 | Middle complexity | 209 | 7 |
| Oncology | RBI 2 | Lower complexity | 227.5 | 8 |
| Oncology | RBI 3-5 | Higher complexity | 242 | 9 |
| Oncology | RBI 3-5 | Middle complexity | 237 | 25 |
| Oncology | RBI 3-5 | Lower complexity | 200.5 | 46 |

S5 Table. Counterfactual model, bootstrap outputs, and product-level predictions

Section A. Counterfactual scenario summary

| Scenario | Neurology products, n | Observed median RBI | Counterfactual RBI | Observed predicted RAT, days | Counterfactual predicted RAT, days | Median days saved [IQR] | Range of days saved | Bootstrap median days saved [95% interval] |
| --- | --- | --- | --- | --- | --- | --- | --- | --- |
| Raise neurology RBI to oncology median (RBI = 3) | 59 | 2 | 3 | 346.6 | 289.8 | 37.8 [0.0–115.0] | 0.0–133.2 | 38.1 [22.1–55.5] |
| Raise neurology RBI to oncology upper quartile (RBI = 4) | 59 | 2 | 4 | 346.6 | 262.2 | 71.5 [32.8–145.3] | 0.0–168.3 | 70.7 [40.8–101.7] |

Section B. Counterfactual model fit

| Metric | Value |
| --- | --- |
| R² | 0.191 |
| Adjusted R² | 0.170 |
| Residual standard error (sigma) | 0.492 |
| Model statistic | 8.94 |
| Model p-value | <0.001 |
| Degrees of freedom | 5 |
| Residual degrees of freedom | 189 |
| Log likelihood | −135.19 |
| AIC | 284.39 |
| BIC | 307.30 |
| Deviance | 45.68 |
| Observations, n | 195 |

Section C. Counterfactual model coefficients

| Model term | Estimate | Standard error | Statistic | p-value | 95% CI |
| --- | --- | --- | --- | --- | --- |
| Intercept | 6.182 | 0.295 | 20.96 | <0.001 | 5.601 to 6.764 |
| Oncology therapeutic area | −0.143 | 0.093 | −1.54 | 0.125 | −0.326 to 0.040 |
| Regulatory Bundling Index | −0.115 | 0.028 | −4.14 | <0.001 | −0.171 to −0.060 |
| Product-level evidence complexity | 0.047 | 0.033 | 1.45 | 0.149 | −0.017 to 0.111 |
| Log enrollment | −0.066 | 0.045 | −1.47 | 0.144 | −0.154 to 0.023 |
| Product-level curated NCT links | 0.029 | 0.056 | 0.53 | 0.598 | −0.080 to 0.139 |

Section D. Product-level counterfactual predictions

The full product-level prediction dataset is provided in the spreadsheet version of this table. For each neurology product, the dataset reports the observed RBI, counterfactual RBI, observed predicted RAT, counterfactual predicted RAT, estimated days saved, and observed RAT. This section supports the paired product-level counterfactual visualization shown in S5 Fig.

S6 Table. Economic translation outputs

| Scenario | Neurology products, n | Observed median RAT cost | Counterfactual median RAT cost | Median RAT-cost saving | Median CDT cost held constant | Observed median total cost | Counterfactual median total cost | Median total-cost saving [IQR] |
| --- | --- | --- | --- | --- | --- | --- | --- | --- |
| Raise neurology RBI to oncology median (RBI = 3) | 59 | $346.6M | $289.8M | $37.8M | $1.19B | $1.60B | $1.50B | $37.8M [$0.0M–$115.0M] |
| Raise neurology RBI to oncology upper quartile (RBI = 4) | 59 | $346.6M | $262.2M | $71.5M | $1.19B | $1.60B | $1.47B | $71.5M [$32.8M–$145.3M] |

S7 Table. Supplementary predictive sensitivity analysis

| n_products | folds | outcome | r2_log_scale | rmse_days | mae_days | median_absolute_error_days | |  |
| --- | --- | --- | --- | --- | --- | --- | --- | --- |
| 195 | 5 | log(RAT_days), back-transformed to days for RMSE and MAE | 0.02887 | 357.4657 | 137.1637 | 72.44686 |  |  |

S8 Table. Robustness analyses

| check | p_value | p_value_fmt |
| --- | --- | --- |
| RAT by area - full cohort | 4.26E-09 | p < 0.001 |
| RAT by area - products with valid NCT only | 1.29E-08 | p < 0.001 |
| RAT by area - excluding top 1% RAT | 4.44E-09 | p < 0.001 |
| Winsorized RAT by area | 4.30E-09 | p < 0.001 |
| RBI by area - full cohort | 1.26E-06 | p < 0.001 |
| CDT by area - full cohort | 0.703059 | p = 0.703 |
| TTA by area - full cohort | 0.412778 | p = 0.413 |

S9 Table. Exploratory design-complexity score components

| Component | Derived variable / source field | Coding in score | Score contribution | Rationale |
| --- | --- | --- | --- | --- |
| Randomized allocation | Allocation status from exact AACT-enriched pivotal trial record | Randomized = 1; not randomized = 0 | 0 or 1 | Randomized allocation reflects a more structured pivotal evidence architecture than non-randomized evidence. |
| Masking | Masking status from exact AACT-enriched pivotal trial record | Masked = 1; not masked/open-label = 0 | 0 or 1 | Masking increases trial-control structure and implementation complexity. |
| Late-phase status | Trial phase from exact AACT-enriched pivotal trial record | Phase 3 or phase 2/3 = 1; other phase categories = 0 | 0 or 1 | Late-phase studies generally represent confirmatory or near-confirmatory evidence and require more structured trial execution. |
| Intervention model | Intervention model from exact AACT-enriched pivotal trial record | Non-single-group design = 1; single-group design = 0 | 0 or 1 | Comparator or multi-group designs generally require more complex trial conduct than single-group evidence. |
| Enrollment scale | Enrollment value from exact AACT-enriched pivotal trial record | min(log10(enrollment + 1) / 4, 1) | 0 to 1 | Log-transformed enrollment captures trial scale while limiting the influence of very large trials. |
| Overall exploratory design-complexity score | Sum of the five components above | Randomized allocation + masking + late-phase status + non-single-group design + enrollment scale | 0 to 5 | Higher values indicate more structured pivotal evidence architecture. The score was used descriptively and was not treated as a validated clinical-development burden instrument. |

S10 Table. Abbreviations

| Abbreviation | Definition |
| --- | --- |
| AA | Accelerated Approval |
| AACT | Aggregate Analysis of ClinicalTrials.gov |
| BLA | Biologics License Application |
| BTD | Breakthrough Therapy designation |
| CDT | Clinical development time |
| CI | Confidence interval |
| FDA | U.S. Food and Drug Administration |
| FT | Fast Track |
| IQR | Interquartile range |
| NCT | National Clinical Trial identifier |
| NDA | New Drug Application |
| ORP | Orphan designation |
| PR | Priority Review |
| RAT | Regulatory approval time |
| RBI | Regulatory Bundling Index |
| TTA | Total time to approval |
| QALY | Quality-adjusted life-year |
| ICER | Incremental cost-effectiveness ratio |

**SUPPLEMENTARY FIGURES**

S1a.

S1b.

**S1 Fig. Curated linkage and source-coverage audit.** A. shows the FDA-source-anchored denominator flow, including the final product-level cohort, products with valid curated NCT identifiers, product–NCT pivotal links, distinct NCT identifiers, and exact AACT-matched links. B. shows source coverage by therapeutic area, distinguishing products with valid curated NCT identifiers from products retained only in product-level regulatory analyses.

2a.

2b.

2c.

2d.

**S2 Fig. Expanded pivotal evidence architecture by therapeutic area.** Panel A shows allocation status among curated product–NCT pivotal links. Panel B shows masking status. Panel C shows trial phase group. Panel D shows intervention model. Percentages are calculated at the product–NCT link level within each therapeutic area.

**3a.**

**3b.**

**S3 Fig. Curated pivotal-trial linkage and enrollment distributions.** Panel A shows the number of curated pivotal NCT links per product by therapeutic area. Panel B shows enrollment distributions among exact NCT-enriched pivotal links. Boxplots show median and interquartile range, with individual points representing products or product–NCT links as appropriate.

**S4a.**

**S4b.**

**S4 Fig. Robustness of regulatory approval time differences.** Panel A compares regulatory approval time by therapeutic area in the full cohort and in the subset of products with valid curated NCT identifiers. Panel B shows the regulatory approval time comparison after outlier handling, including winsorized analyses. These analyses assess whether the primary regulatory approval time difference is sensitive to source coverage or extreme values.

**S5 Fig. Product-level counterfactual predicted regulatory approval time pairs. Each line represents one neurology product.** The left point shows predicted regulatory approval time under the observed pathway profile, and the right point shows predicted regulatory approval time after pathway intensification to the oncology-median Regulatory Bundling Index scenario.

**S6a.**

**S6b.**

**S6 Fig. Supplementary predictive sensitivity analysis.** Panel A shows calibration of the supplementary gradient-boosted model for log-transformed regulatory approval time. Panel B shows feature importance from the same model. This analysis was performed only as a supplementary predictive sensitivity analysis and was not used for primary inference.
